# Suppressive Effects of Tea Catechins on Breast Cancer

**DOI:** 10.3390/nu8080458

**Published:** 2016-07-28

**Authors:** Li-Ping Xiang, Ao Wang, Jian-Hui Ye, Xin-Qiang Zheng, Curt Anthony Polito, Jian-Liang Lu, Qing-Sheng Li, Yue-Rong Liang

**Affiliations:** 1 Tea Research Institute, Zhejiang University, # 866 Yuhangtang Road, Hangzhou 310058, China; gzzyzj_2009@vip.sina.com (L.-P.X.); jianhuiye@zju.edu.cn (J.-H.Y.); xqzheng@zju.edu.cn (X.-Q.Z.); curtpolito@outlook.com (C.A.P.); jllu@zju.edu.cn (L.-J.L.); qsli@zju.edu.cn (Q.-S.L.); 2 National Tea and Tea product Quality Supervision and Inspection Center (Guizhou), Zunyi 563100, China; wangaocn@gmail.com

**Keywords:** *Camellia sinensis*, anticancer, antioxidant, signaling pathway, anti-proliferation, DNA methylation, metastasis

## Abstract

Tea leaf (*Camellia sinensis*) is rich in catechins, which endow tea with various health benefits. There are more than ten catechin compounds in tea, among which epigallocatechingallate (EGCG) is the most abundant. Epidemiological studies on the association between tea consumption and the risk of breast cancer were summarized, and the inhibitory effects of tea catechins on breast cancer, with EGCG as a representative compound, were reviewed in the present paper. The controversial results regarding the role of tea in breast cancer and areas for further study were discussed.

## 1. Introduction

Breast cancer is a common cancer in women. There were an estimated 1.7 million new cases (25% of all cancers in women) and 0.5 million cancer deaths (15% of all cancer deaths in women) in 2012 [1]. Though there have been great advances in the treatment of breast cancer, mortality from breast cancer is still high, and it is the second leading cause of cancer-related death among women in the United States [2]. Diet is considered to be an important factor preventing breast cancer [2,3].

Tea is one of the most popular beverages consumed all over the world. Tea leaves are rich in catechins, a group of polyphenols that endow tea with many health benefits. (−)-Epigallocatechingallate (EGCG), (−)-epicatechingallate (ECG), (−)-epigallocatechin (EGC), and (−)-epicatechin (EC) are the major catechins in fresh tea leaf, while more than ten catechins are usually detected in various kinds of processed teas, owing to the isomerization of epi-type catechins during tea processing [4]. Teas are classified into fully-fermented black tea, semi-fermented Oolong tea, and unfermented green tea, based on the degree of fermentation, during which catechins are oxidized. EGCG is the most abundant catechin in tea, and it accounts for more than 40% of total catechins in green tea leaves [5]. The total concentration of catechins is 58.0–183.9 mg/g in green tea [4], 74.8–105.7 mg/g in oolong tea [6], and 11.7–55.3 mg/g in black tea [7]. Green tea polyphenols (GTP) are considered to be a potential candidate for further development as a chemoprotective factor for the primary prevention of age-related eye diseases [8]. There have been epidemiological and in vitro studies regarding the association of tea consumption with depression among breast cancer survivors [9]. Drinking tea or green tea was not associated with overall breast cancer risk [10]. However, the effects of tea and its catechins on the prevention of breast cancer are still inconclusive and controversial [11,12]. The present review will highlight the recent advances in the effects of tea and its catechins on breast cancer, including epidemiological, in vivo, and in vitro studies. The controversial results from in vitro and in vivo studies, as well as directions for further study are also discussed in the present paper.

## 2. Epidemiological Evidence

As early as 1997, an epidemiological study carried out in Japan showed that drinking green tea had a potentially preventive effect on breast cancer, especially among women who drank more than 10 cups of green tea per day [13]. Many cohort studies or case-control studies on the association between tea consumption and breast cancer risk have been carried out since then. Cohort studies in China and the USA showed that habitual drinking of green tea was weakly associated with a decreased risk of breast cancer [14,15]. There was a time-dependent interaction between green tea consumption and age of breast cancer onset (*p* for interaction = 0.03). Women who started drinking tea at the age of 25 or younger had a hazard ratio (HR) of 0.69 (95% confidence interval (CI): 0.41–1.17) to develop premenopausal breast cancer, compared with non-tea drinkers [16]. Tea drinking was also helpful to the treatment of breast cancer patients. Habitual tea-drinking (more than 100 g dried tea per month) was inversely associated with depression among patients who were diagnosed with stage 0 to III breast cancer, with odds ratio (OR) 0.39 and 95% CI ranging from 0.19 to 0.84 [9]. Drinking tea or green tea was not associated with overall breast cancer risk [10].

The association between tea consumption and decreased risk of breast cancer was also confirmed by population-based case-control studies carried out in China [10,12,17], the USA [18,19,20], and Singapore [21,22]. There were studies showing that green tea consumption significantly reduced the risk of breast cancer [18] and the women who drank three or more cups of tea per day had a 37% reduced breast cancer risk than their counterparts that did not drink tea [20]. Differences in the efficacy of tea consumption on breast cancer were observed between various populations. Among women with high-activity of the angiotensin-converting enzyme (ACE) genotype, green tea intake frequency significantly decreased the risk of breast cancer (*p* = 0.039) [21]. Among women with low folate intake or high-activity MTHFR/TYMS (methylene tetrahydrofolate reductase /thymidylate synthetase) genotypes, green tea consumption was inversely associated with breast cancer risk [22], suggesting that folate pathway inhibition might be one of the mechanisms for the protection that green tea provides against breast cancer in humans. A significant association between regular tea consumption and lower risk for breast cancer [12] was observed among premenopausal Chinese women (OR = 0.62, 95% CI: 0.40–0.97) [10], but an increased risk was seen in postmenopausal women (OR = 1.40, 95% CI: 1.00–1.96) [10]. An inverse association between tea consumption and breast cancer was observed among younger women (less than 50 years old), which was consistent for in situ and invasive breast cancer and ductal and lobular breast cancer [20]. Combined intake of green tea and mushroom showed an additional decreased risk of breast cancer [17]. Table 1 lists the epidemiological evidence for the association between tea intake and the risk of breast cancer.

## 3. Mechanism of Tea Catechins in Suppressing Breast Cancer

### 3.1. Suppressing Carcinogen-Induced ROS Elevation and DNA Damage

ROS (reactive oxygen species) are a group of chemically-reactive molecules, including hydrogen peroxide, superoxide anion radical, singlet oxygen, and hydroxyl radicals, which are crucially involved in multiple stages of carcinogenesis [24]. The anti-carcinogenic activity of tea catechins is considered to be related to their protection of DNA from ROS-induced damages by alleviating ROS stress. It was shown that short-term exposure of breast cancer cells to 4-(methylnitrosamino)-1-(3-pyridyl)-1-butanone (NNK) and benzo[a]pyrene (B[a]P) would increase the level of ROS, resulting in the activation of the extracellular signal-regulated kinase (ERK) pathway and subsequent induction of DNA damage [25]. In vitro [26] and in vivo [27] studies showed that tea catechins prevented breast carcinogenesis by alleviating ROS stress. Ten μg/mL EGCG suppressed chronically-induced cellular carcinogenesis by blocking carcinogen-induced ROS elevation [25]. Tea polyphenols, such as green tea catechins and black tea theaflavins, could inhibit DNA cleavage induced by the combination of hydrogen peroxide and cytochrome c [26]. Green tea catechins or black tea theaflavins delay mammary carcinogenesis in the TAg mouse model, which is accompanied by an antioxidant effect in the target organ, as reflected by levels of M1dG (malondialdehyde–deoxyganosine) adducts [27] which is a prevalent guanine adduct formed by a condensation reaction between guanosine. Furthermore, the combination of catechins with anticancer drugs such as tamoxifen (TAM) showed an etiological role in the abrogation of TAM-induced toxicity by relieving oxidative stress and biochemical perturbations [28].

The mechanism for the alleviation of ROS stress by catechins includes their increasing of the activity of anti-oxidases such as catalase, superoxide dismutase (SOD), and glutathione peroxidase (GHS-px) [29] directly scavenging ROS [25], preventing the iron-induced generation of hydroxyl free radicals via Haber–Weiss and Fenton reactions by chelating ferrous iron. The potent antioxidant and anti-inflammatory activities of EGCG are also beneficial to the modulation of mitochondrial functions, impacting mitochondrial bioenergetic control, cell cycle, and mitochondria-related apoptosis [30].

### 3.2. Regulating Cell Signaling Pathways

The PI3K/Akt/mTOR (phosphoinositide-3-kinase/protein kinase B/mammalian target of rapamycin) signaling pathway is a commonly activated signaling pathway in human cancer. The important nodes in this pathway are used as key therapeutic targets for cancer treatments. EGCG was confirmed to be an ATP-competitive inhibitor of both PI3K and mTOR in breast cancer cells MDA-MB-231, with *Ki* values ranging 380 nM to 320 nM, respectively [30]. Molecular docking studies showed that EGCG binds well to the PI3K kinase domain active site, showing ATP-competitive activity [31]. Tumor-associated fatty acid synthase (FAS) is implicated in breast carcinoma and is connected to the epidermal growth factor receptor (EGFR) signaling pathway. Suppression of FAS in cancer cells may lead to growth inhibition and the apoptosis of breast cancer cells. EGCG suppressed EGFR signaling and downstream phosphatidylinositol 3-kinase (PI3K)/Akt activation in the MCF-7 breast cancer cell line, resulting in down-regulation of FAS expression. It is considered that EGCG may be useful in the chemoprevention of breast carcinoma in which FAS over-expression results from signaling of human epidermal growth factor receptor 2 (HER2) or/and HER3, two members of EGFR family [32]. Exposure to carcinogens such as 4-(methylnitrosamino)-1-(3-pyridyl)-1-butanone (NNK) and benzo[a]pyrene (B[a]P) will result in an elevation of ROS, leading to activation of the Raf-independent extracellular signal-regulated kinase (ERK) pathway, which will induce DNA damage. Green tea extract (GTE) was confirmed to inhibit the activation of the ERK pathway by blocking carcinogen-induced ROS elevation, resulting in the suppression of chronically-induced breast cell carcinogenesis [25]. Wnt (wingless integrated) proteins are a group of highly conserved secreted signaling molecules which play critical roles during embryonic development and in the regeneration of adult tissues. Mutations in Wnt genes or Wnt pathway components lead to developmental defects and many cancers are caused by abnormal Wnt signaling. EGCG induced HMG-box transcription factor 1 (HBP1) transcriptional repressor, resulting in blockage of the Wnt/β-catenin pathway and inhibition of both breast cancer cell tumorigenic proliferation and invasiveness [33]. Met, a hepatocyte growth factor (HGF) receptor, is a strong prognostic indicator of breast cancer patient outcome and survival. Therapies targeting Met will have beneficial clinic outcomes. Catechins with R1 galloyl and R2 hydroxyl groups had a strong ability to inhibit HGF/Met signaling and block invasive breast cancer [34].

### 3.3. Interacting with Target Proteins

Estrogen is associated with the initiation and growth of breast cancer due to its action on proto-oncogenes and breast cell proliferation [35]. The interactions between estrogen and its specific estrogen receptor (ERs) proteins are increasingly drawing research interest in breast cancer etiology and clinical therapy studies. The ERs are classified into nuclear ERs and membrane ERs [36,37]. ERα and ERβ are two important subtypes of nuclear ERs, and they are used as reference for clinical diagnosis and therapy decisions regarding breast cancer [35,37]. Synthetic ER antagonists were designed to occupy the ligand-binding pocket to block the access of estrogen to the ERs, which have been used clinically in the treatment of ER-positive breast cancer [37]. The interaction between catechins and ERs showed anti-estrogenic activity, and so catechins are considered for use as potential phytoestrogens to replace synthetic ER antagonists in clinical use [32,38,39]. EGCG could reactivate ERα expression in ERα-negative breast cancer cells by its remodeling effect on the chromatin structure of the ERα promoter through altering histone acetylation and methylation status [40]. These results support further preclinical and clinical evaluation of EGCG as a therapeutic option for ER-negative breast cancer. Furthermore, EGCG can bind with high affinity to many other target proteins in cancer cells, such as 70 kDa zeta-associated protein (Zap-70) [41], 67-kDa laminin receptor [42], phosphoinositide 3 kinase (PI3K) [31], Ras-GTPase activating protein (GAP), SH3 domain-binding protein 1 (G3BP1) [43], insulin-like growth factor 1 receptor (IGF-1R) [44], vimentin [45], Bcl-2 and Bcl-xL [46], GRP78 [47], and Fyn [48], resulting in the inhibition of breast cancer. EGCG interacts with target proteins via hydrogen bonding, during which the hydroxyl groups of EGCG serve as hydrogen bond donors.

### 3.4. Inhibiting DNA Methylation

DNA methylation is an important epigenetic mechanism for the inactivation of many genes related to tumor suppressors and DNA repair enzymes [49]. DNA methylation is catalyzed by specific DNA methyltransferase (DNMT) or catechol-*O-*methyltransferase (COMT), in which *S*-adenosyl-l-methionine (SAM) is the methyl donor. *S*-adenosyl-l-homocysteine (SAH)—a potent noncompetitive inhibitor of DNMTs—is formed when the methyl group of SAM combines with the DNA substrate. Recent studies showed that tea catechins inhibited human DNMT-mediated DNA methylation through two mechanisms—i.e., the direct inhibition of DNMTs by catechins and the indirect inhibition of DNMTs by increasing the SAH level. Their inhibitory potency is in the rank order of EGCG > ECG > EGC > EC, based on the concentration for 50% inhibition (IC_50_) [50]. EGCG interacted with DNMT enzyme by forming hydrogen bonds with proline^1223^, glutamate^1265^, cysteine^1225^, serine^1229^, and arginine^1309^ in the catalytic pocket of DNMT, and the B and D ring moieties of EGCG played important roles [51]. Synthetic analog of EGCG had the same effect on COMT as EGCG [52]. Methylated EGCG led to decreased proteasome-inhibitory activity and cancer-preventive effects of EGCG [53]. The suppressive effects of EGCG on DNA methylation were closely associated with its anti-tumor activity [54].

### 3.5. Inhibiting Tumor Angiogenesis

Angiogenesis, which is essential for tumor growth, can provide nutrients and oxygen for tumor growth [55]. Vascular endothelial growth factor (VEGF), the most effective angiogenesis factor, has been reported to stimulate endothelial cells in the proliferation of tumor blood vessels. Subsequently, the proliferation of endothelial cells prompts the formation of new blood vessels [55,56]. Thus, inhibiting angiogenesis would be conducive to tumor suppression. Tea catechins—especially EGCG—exert prominent antiangiogenic activity [57,58,59], which results in decreased breast cancer risk. Catechins could effectively inhibit VEGF expression in breast cancer cells, leading to the suppression of endothelial cell formation and angiogenesis. GTE or EGCG suppressed VEGF protein secretion by inhibiting VEGF promoter activity, resulting in decreased expression of VEGF transcript, c-jun transcript, c-fos transcript, and protein kinase C (PKC) [60].

The antiangiogenic mechanism of catechins is closely related to VEGF signaling intervention. The VEGF-induced angiogenesis signal pathway is initiated through a multi-component receptor complex composed of VEGF-2, β-bcatenin, VE-cadherin, and PI3-kinase. Catechins inhibited the formation of the multi-component receptor complex, resulting in the interference of VEGF signaling and the inhibition of endothelial cell formation [61].

### 3.6. Anti-Proliferation and Inducing Breast Cancer Cell Apoptosis

The mechanism of action of anticancer drugs is based on their ability to induce apoptosis in cancer cells. Tea catechins such as EGCG, gallocatechin gallate (GCG), and gallocatechin (GC) showed 100%, 97%, and 95% inhibition of breast cancer cell proliferation, respectively at a concentration of 50 μM, [62]. Tea catechins suppress proliferation and induce apoptosis of breast cancer cells via several pathways, including: (1) Inducing cell cycle arrest. The cell growth of human breast cancer cell line T47D was arrested at the G(2)/M phase in a dose-dependent manner by EGCG. The mechanism is that catechins phosphorylate c-jun N-terminal kinase/stress activated protein kinase (JNK/SAPK) and p38. The phosphorylated JNK/SAPK and p38 inhibit the phosphorylation of cell division cycle 2 (cdc2) and regulate the expression of cyclin A, cyclin B1, and cyclin-dependent kinase proteins, resulting in G(2) arrest [63]; (2) Promoting tumor protein P53 (TP53)/caspase-mediated apoptosis. Catechin hydrate (CH) is a strong antioxidant and an efficient scavenger of free radicals. CH exhibits anticancer effects by inhibiting the proliferation of breast cancer cells and inducing the apoptosis of cancer cells, partially through suppression of the expression of caspase-3, caspase-8, caspase-9, and TP53 [64]; (3) Down-regulating anti-apoptotic factors. Catechins such as catechin, GC, and catechin gallate (CG) induced breast cancer cell apoptosis by suppressing the expression of anti-apoptotic factors such as B cell lymphoma 2 (Bcl-2), Bcl-xL, and survivin, accompanied by the inhibition of NFκB, JAK/STAT, and PI3K pathways [65]. Oligonol—a catechin-rich preparation—triggered apoptosis in estrogen-responsive MCF-7 and estrogen-unresponsive MDA-MB-231 breast cancer cells through the modulation of pro-apoptotic Bcl-2 family proteins and MEK/ERK signaling pathway [66]; (4) Inhibiting fatty acid synthase (FAS). FAS is a breast cancer-associated enzyme connected to human epidermal growth factor receptor (HER). Suppression of FAS may lead to cancer cell apoptosis. EGCG down-regulated FAS by suppressing HER2 or/and HER3 signaling and downstream PI3K/Akt activation in the MCF-7 breast cancer cell line [32]; (5) Regulating NO/NOS system. Catechins (10^−7^ M) inhibited proliferation of human breast cancer cell T47D, with cells being arrested at the S phase of cell cycle. The anti-proliferative activity of catechins is considered to be involved in the nitric oxide/nitric oxide synthase (NO/NOS) system because catechin treatment decreased NOS, resulting in NO reduction [67]. However, the role of NO in regulating the anti-proliferative effect of catechins is ambiguous, and the regulation mechanism of catechins on the NO/NOS system is not fully clear yet; (6) Inducing Ca^2+^-associated apoptosis. EGCG induced an increase in endoplasmic reticulum calcium ([Ca^2+^]_ER_) and a decrease in cytosolic Ca^2+^ by inhibiting Bcl-2 mediated Ca^2+^ leakage from the endoplasmic reticulum in MCF-7 breast cancer cells [68]. EGCG acts via the signaling pathways related to cell membrane and endoplasmic reticulum stress to suppress cell proliferation or provoke apoptosis [69].

### 3.7. Anti-Metastasis of Breast Cancer Cells

The metastasis of cancer cells includes three key steps; i.e., adhesion, migration, and invasion. EGCG can effectively inhibit the invasion and migration of breast cancer cells, resulting in decreased lung and liver metastasis [70]. Tea catechins showed an inhibitory effect on the migratory and invasive potential of breast cancer cells [71,72]. Catechins inhibit metastasis by modulating the activity of proteolytic enzymes, regulating the signaling pathway and growth factor/receptor, suppressing the epithelial-to-mesenchymal transition (EMT) process and inhibiting angiogenesis [72,73].

Degradation of extracellular matrix components by matrix metalloproteinases (MMPs) and other proteolytic enzymes is critical in tumor invasion and metastasis behavior. Pro-MMP-2 is a proenzyme involved in the malignant progression of tumors. Membrane type-1 matrix metalloproteinase (MT1-MMP) cleaves the N-terminal prodomain of pro-MMP-2, which generates the active intermediate that is modified into the fully active enzyme MMP-2 afterwards. EGCG down-regulated MT1-MMP transcription, resulting in the inhibition of the MT1-MMP-driven migration of breast cancer cells [73]. Catechins can modulate the secretion of urokinase plasminogen activator (uPA), which is closely related to proteolytic enzymes in breast cancer cells and inhibits their invasive behavior by suppressing the transcription factors AP-1 and NF-κB [71]. EGCG can also remarkably attenuate lipopolysaccharide (LPS)-induced cell migration by a significant internalization of 67KD laminin receptor (67LR) [42,73].

EGCG also plays important roles in inhibiting tumor metastasis through the modulation of signaling pathways, including the modulation of β1 integrin-mediated signaling [74], down regulation of vasodilator-stimulated phosphoprotein (VASP) expression via the Rac1 pathway [72], enhancing the expression of α1-antitrypsin by regulating the PI3K/AKT pathway [75], and down-regulating the EGFR signaling pathway [76].

## 4. Inconsistent Results and Further Study Suggestions

### 4.1. Inconsistent Results

Although animal and in vitro studies showed that tea catechins were associated with a protective role against breast cancer, evidence from in vivo and human epidemiological studies is inconsistent. Increased green tea consumption (>3 cups/day) was inversely associated with recurrence (Pooled RR = 0.73, 95% CI: 0.56–0.96). An analysis of case–control studies investigating incidence suggested an inverse association, with a pooled RR of 0.81 (95% CI: 0.75, 0.88), while no association was found among cohort studies of incidence [23,77,78]. There are many factors leading to the controversial results.

First, the suppressive effects of tea on breast cancer differed between various kinds of tea. The reduction in breast cancer risk was usually associated with green tea consumption, rather than black tea consumption [10,11,15,17,79]. The major bioactive components in tea are catechins, especially EGCG. Black tea is a fully-fermented tea, and about 80% of tea catechins are oxidized and converted into orange and red tea pigments (theaflavins and thearubigins) during fermentation. This may explain why black tea consumption was not associated with the decreased risk of breast cancer.

Second, contradictory results arose from different populations investigated. An epidemiological study showed that daily tea consumption was significantly associated with a lower risk in the population of premenopausal women (OR = 0.62, 95% CI: 0.40–0.97), but an increased risk for breast cancer in the population of postmenopausal women (OR = 1.40, 95% CI: 1.00–1.96). The relationship between drinking green tea with the risk of breast cancer differed between ER-negative (OR = 1.22, 95% CI: 0.43–3.43) and ER-positive (OR = 0.61, 95% CI: 0.25–1.49) populations among postmenopausal women [10]. The observations of men and women gave different results. Tea drinking showed a strong association with increased risk for breast cancer in men, but no association with the development of breast cancer in women [80]. There was a significant association between the intake frequency of green tea and the decrease in risk of breast cancer among women with high-activity of the angiotensin-converting enzyme (ACE) genotype. However, no association was observed between the intake frequency of green tea and the risk of breast cancer among women with the low-activity ACE genotype [21]. These controversial results might be due to the differences in physiological status between various populations, which gave different responses to the bioactive components in tea.

Third, contradictory results between in vitro and in vivo studies arose from low bioavailability and biotransformation in vivo. When EGCG was incubated with rat liver microsomes at 1–100 μM for 30 min in vitro, EGCG selectively bound to COMT [81]. However, in vivo tests showed that supplementation with a high dose of EGCG does not impair the activity of COMT [82]. A bioavailability test using ^3^H-EGCG in mice revealed a wide distribution of radioactivity in target organs, including digestive tract, liver, lung, pancreas, mammary gland, brain, kidney, uterus, and ovary. However, radioactivity in the blood was low, being about 2% of total administered radioactivity at 6 h after administration, and the status was sustained for 24 h. However, 37.1% of total administered radioactivity was excreted in feces and 6.6% in urine within 24 h [83]. Chemical modification of tea catechins occurring in the digestive tract might lead to their low bioavailability. Under physiological conditions, COMT can metabolize EGCG to 4″-o-methyl-EGCG (MeEGCG) and 4′,4″-di-o-methyl-EGCG (DiMeEGCG), resulting in a reduction of the oral bioavailability of EGCG and reduced cancer-related biological activities of EGCG. Combination of EGCG and Tolcapone (TOL) (a COMT inhibitor) was found to improve the bioavailability of EGCG and to synergistically enhance the cancer suppressive effect of EGCG by inhibiting the COMT-mediated methylation of EGCG in vivo [84]. The authors deduced that the differences in the suppressive effect of catechins on breast cancer between various populations might be related to the differentiation in the bioavailability of catechins between different populations, owing to variations in physiological status.

### 4.2. Further Study Suggestions

Improvement of the bioavailability of the bioactive catechins will be an important research topic in the future. The development of methods to improve the stability of tea catechins will enhance their oral bioavailability. The usage of stabilizers and/or encapsulation of EGCG into particulate systems such as nanoparticles or microparticles can significantly increase its stability [85]. It was reported that encapsulation of tea extract in chitosan encapsulation in nanoparticles (NPs) was beneficial in stabilizing catechins including EGCG and catechin (C) in vivo, resulting in a significant improvement of their intestinal absorption [86]. Encapsulation of catechin and epicatechin (EC) in bovine serum albumin NPs (BSA-NPs) could also improve their stability and antioxidant potential in cell line A549 [87]. Antitumor activity of folate-conjugated chitosan-coated EGCG NPs (FCS-EGCG-NPs)—prepared by ionic gelation method using folic acid-modified carboxymethyl chitosan—gave a greater tumor inhibitory effect on cancer cells than free EGCG, especially in the cancer cells with a strong expression of folic acid receptors on the cell surface [88]. Loading EGCG in cationic lipid nanoparticles (LNs) is recognized as a promising strategy for prolonging EGCG release [89].

Developing complex formulations using various tea catechins and other bioactive components will also improve the stability and bioavailability of tea catechins. Although EC did not induce apoptosis of lung cancer cell line PC-9, co-treatment of EGCG with 100 μM EC reduced the IC_50_ of EGCG from 60 μM to 15 μM, suggesting that EC enhanced the anti-cancer activity of EGCG [83]. The combination of 75 μM EGCG with the cancer preventive agent Sulindac (10 μM or 100 μM) induced apoptosis of PC-9 cells over 10 times more strongly than Sulindac alone [83]. The cellular accumulation of EC was increased by co-administrating with other catechins, especially gallated catechins [90]. Green tea catechins, formulated with xylitol and vitamin C and then encapsulated in g-cyclodextrin (g-CD) or coated with hydroxypropyl methyl cellulose phthalate (HPMCP), provided a synergistic effect to significantly enhance the intestinal absorption of catechins [91]. Encapsulation of hydrophilic catechin and hydrophobic curcumin within a water-in-oil-in-water (W/O/W) double emulsion by a two-step emulsification method significantly increased their stability in simulated gastrointestinal fluid and gave a four-fold augmentation in their bio-accessibility, compared to that of freely-suspended curcumin and catechin solutions [92]. When EGCG was loaded into hydrogel prepared by ionic interaction gelatin and γ-polyglutamic acid with ethylcarbodiimide as the crosslinker, EGCG was more stable in the harsh gastrointestinal tract environment than free EGCG [93].

However, encapsulated EGCG should be taken without food in order to maximize its systemic absorption, because the co-administration of EGCG with foods such as a light breakfast or strawberry sorbet reduced systemic or plasma EGCG [94].

## 5. Conclusions

**Figure 1:**
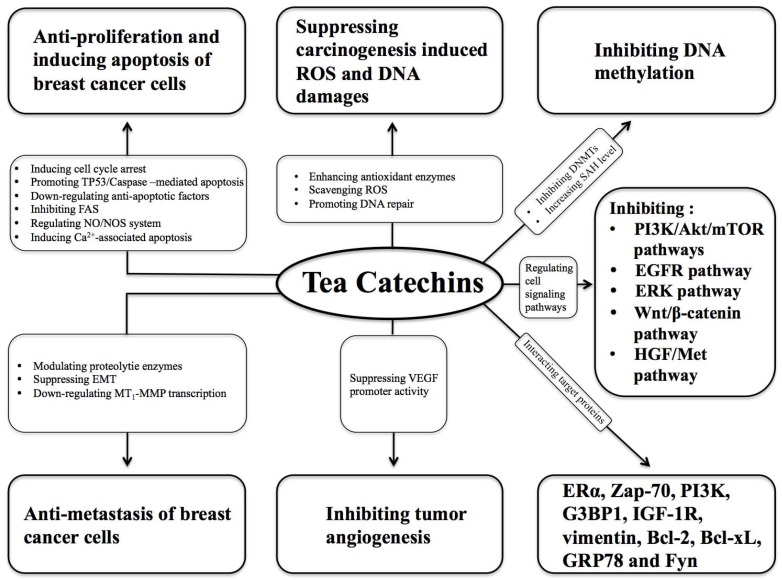
Effects of tea catechins on breast cancer. Akt: protein kinase B; DNMT: DNA methyltransferase; EGFR: epidermal growth factor receptor; EMT: epithelial-to-mesenchymal transition; ERα: estrogen receptor alpha; ERK: extracellular signal-regulated kinase; FAS: fatty acid synthase; G3BP1: SH3 domain-binding protein 1; HGF: hepatocyte growth factor; IGF-1R: insulin-like growth factor 1 receptor; MT1-MMP: membrane type-1 matrix metalloproteinase; mTOR: mammalian target of rapamycin; NO/NOS: nitric oxide/nitric oxide synthase; PI3K: phosphoinositide-3-kinase; ROS: reactive oxygen species; SAH: *S*-adenosyl-L-homocysteine; TP53: tumor protein P53; VEGF: vascular endothelial growth factor; Zap-70: 70 kDa zeta-associated protein.

Though the role of tea in breast cancer is uncertain, there have been many in vitro and in vivo studies showing the association between green tea consumption and the decreased risk of breast cancer. There are more than ten catechin compounds in tea, among which EGCG is the most abundant and shows the most active suppressing effects on breast cancer. Catechins are a group of natural antioxidants, and they suppress carcinogen-induced ROS and DNA damage by enhancing antioxidant enzymes, scavenging ROS, and promoting the repair of damaged DNA. Catechins such as EGCG regulate cell signaling pathways relating to breast carcinogenesis, such as PI3k/Akt/mTOR, EGFR, ERK, Wnt/β-catenin, and HGF/Met pathways. EGCG interacts with target proteins in the breast cancer cells, such as ERα, Zap-70, PI3K, G3BP1, IGF-1R, vimentin, Bcl-2, Bcl-xL, GRP78, and Fyn via hydrogen bonding, which plays a role in the inhibition of breast cancer. Catechins inhibit DNA methylation by suppressing DNMTs and increasing SAH levels. GTE or EGCG suppressed the secretion of VEGF protein by inhibiting VEGF promoter activity, resulting in the inhibition of tumor angiogenesis. Catechins suppress proliferation and induce apoptosis of breast cancer cells by inducing cell cycle arrest and Ca^2+^-associated apoptosis, promoting TP53/caspase-mediated apoptosis, down-regulating anti-apoptotic factors, inhibiting FAS, and regulating the NO/NOS system. Tea catechins inhibit metastasis of breast cancer cells via the modulation of proteolytic enzymes, suppressing the EMT, and down-regulating MT1-MMP transcription (Figure 1).

The inconsistent results between in vitro and in vivo studies are considered to arise from the low oral bioavailability and the biotransformation of catechins in vivo. Further studies on the development of methods to stabilize catechins in the digestive tract and complex formulation with synergistic effects between catechins and other ingredients will be beneficial to improve oral bioavailability and anti-tumor effects of tea catechins. Overall, tea catechins show a potential role in suppressing breast cancer.

## Figures and Tables

**Table 1:** Epidemiological evidence for the association between green tea intake and the risk of breast cancer.

Type of Study	Location	Number of Subjects	Main Results	References
Population-based cohort study	Shanghai, China	1399 women with breast cancer	Drinking tea regularly (>100 g dried tea per month) was inversely associated with overall depression.	Chen et al. (2010) [9]
Hospital-based case–control study	Hong Kong, China	Cases: 439 Controls: 434	Habitual tea drinking was significantly associated with a lower risk for breast cancer in premenopausal women (OR = 0.62, 95%CI: 0.40–0.97).	Li et al. (2016) [10]
Case–control study	Southeast China	Cases: 1009 Controls: 1009	Green tea consumption was associated with a reduced risk of breast cancer.	Zhang et al. (2007) [12]
Prospective cohort study	Saitama Prefecture, Japan	9 years of follow-up study (71,248.5 person-years)	Drinking green tea had a potentially preventive effect on breast cancer	Imai et al. (1997) [13]
Population-based study	Shanghai, China	Cases: 3454 Controls: 3474	Drinking green tea regularly was weakly associated with a decreased risk of breast cancer.	Shrubsole et al. (2009) [14]
Long-term cohort study (1980–2002)	Boston, USA	85,987 female participants	There was a significant inverse association of caffeine intake with breast cancer among postmenopausal women	Ganmaa et al. (2008) [15]
Population-based cohort study	Shanghai, China	74,942 Chinese women	Women who started drinking tea at 25 years of age or younger had a hazard ratio 0.69 (CI: 0.41–1.17) to develop premenopausal breast cancer, compared with non-tea drinkers	Dai et al. (2010) [16]
Case–control study	Southeast China	Cases: 1009 Controls: 1009	Green tea intake was associated with decreased breast cancer risk in premenopausal and postmenopausal Chinese women, and there was an additional decreased risk from the joint effect of green tea and mushrooms	Zhang et al. (2009) [17]
Population-based, case–control study	Los Angeles, USA	Cases: 501 Controls: 594	Green tea consumption showed a significantly reduced risk of breast cancer, while black tea consumption was not associated with the risk of breast cancer	Wu et al. (2003) [18]
Population-based case–control study	Massachusetts, USA	Cases: 5082 Controls: 4501	Among women less than 50 years old, those who drank three or more cups of tea per day had a 37% reduced breast cancer risk compared to their counterparts that did not drink tea	Kumar et al. (2009) [20]
Nested case–control study	Singapore	Cases: 297 Controls: 665	There was significant association between green tea intake frequency and decreased risk of breast cancer in the women with high-activity of angiotensin-converting enzyme (ACE) genotype (*p* = 0.039)	Yuan et al. (2005) [21]
Nested case–control study	Singapore	Cases: 380 Controls: 662	Green tea intake was inversely associated with decreased breast cancer risk among women with low folate intake or high-activity MTHFR/TYMS genotypes	Inoue et al. (2008) [22]
Meta-analysis	Boston, USA	Cases: 5617	Increased green tea consumption (>3 cups/day) was inversely associated with recurrence (Pooled RR = 0.73, 95% CI: 0.56–0.96). An analysis of case–control studies of incidence suggested an inverse association with a pooled RR of 0.81 (95% CI: 0.75, 0.88) while no association was found among cohort studies of incidence	Ogunleye et al. (2010) [23]
